# Description of the female, nymph and larva and mitochondrial genome, and redescription of the male of *Ixodes barkeri* Barker, 2019 (Acari: Ixodidae), from the short-beaked echidna, *Tachyglossus aculeatus,* with a consideration of the most suitable subgenus for this tick

**DOI:** 10.1186/s13071-022-05165-2

**Published:** 2022-04-01

**Authors:** Dayana Barker, Samuel Kelava, Renfu Shao, Owen D. Seeman, Malcolm K. Jones, Ryo Nakao, Stephen C. Barker, Dmitry A. Apanaskevich

**Affiliations:** 1 School of Veterinary Sciences, The University of Queensland, Gatton, QLD 4343 Australia; 2 Department of Parasitology, School of Chemistry and Molecular Biosciences, The University of Queensland, Brisbane, QLD 4072 Australia; 3 School of Science and Engineering, GeneCology Research Centre, University of the Sunshine Coast, Sippy Downs, QLD 4558 Australia; 4 Queensland Museum, PO Box 3300, South Brisbane, 4101 Australia; 5 Laboratory of Parasitology, Department of Disease Control, Faculty of Veterinary Medicine, Hokkaido University, Sapporo, 060-0818 Japan; 6 Institute for Coastal Sciences, US National Tick Collection, Georgia Southern University, Statesboro, GA 30460 USA; 7 Department of Biology, Georgia Southern University, Statesboro, GA 30460 USA; 8 Zoological Institute, Russian Academy of Sciences, St. Petersburg, 199034 Russia

**Keywords:** Integrative taxonomy, Wet tropics

## Abstract

**Background:**

*Ixodes barkeri*, a tick with a distinctive ventrolateral horn-like projection on palpal segment 1, was described in 2019 from two male ticks from the Wet Tropics of Far North Queensland, Australia. However, females lie at the core of the taxonomy and subgenus classification of *Ixodes*; hence, we sought specimens of female ticks, successfully recovering females, plus nymphs and larvae. Mitochondrial genomes are also desirable additions to the descriptions of species of ticks particularly regarding subgenus systematics. So, we sequenced the mt genomes of *I. barkeri* Barker, 2019, and the possible relatives of *I. barkeri* that were available to us (*I. australiensis* Neumann, 1904, *I. fecialis* Warburton & Nuttall, 1909, and *I*. *woyliei* Ash et al. 2017) with a view to discovering which if any of the subgenera of *Ixodes* would be most suitable for *I. barkeri* Barker, 2019.

**Results:**

The female, nymph, larva and mitochondrial genome of *Ixodes barkeri* Barker, 2019, are described for the first time and the male of *I. barkeri* is redescribed in greater detail than previously. So far, *I. barkeri* is known only from a monotreme, the short-beaked echidna, *Tachyglossus aculeatus* (Shaw, 1792), from the highland rainforests of the Wet Tropics of Far North Queensland, Australia.

**Conclusions:**

Our phylogeny from entire mitochondrial genomes indicated that *I. barkeri* and indeed *I. woyliei* Ash et al., 2017, another tick that was described recently, are best placed in the subgenus *Endopalpiger* Schulze, 1935.

**Graphical Abstract:**

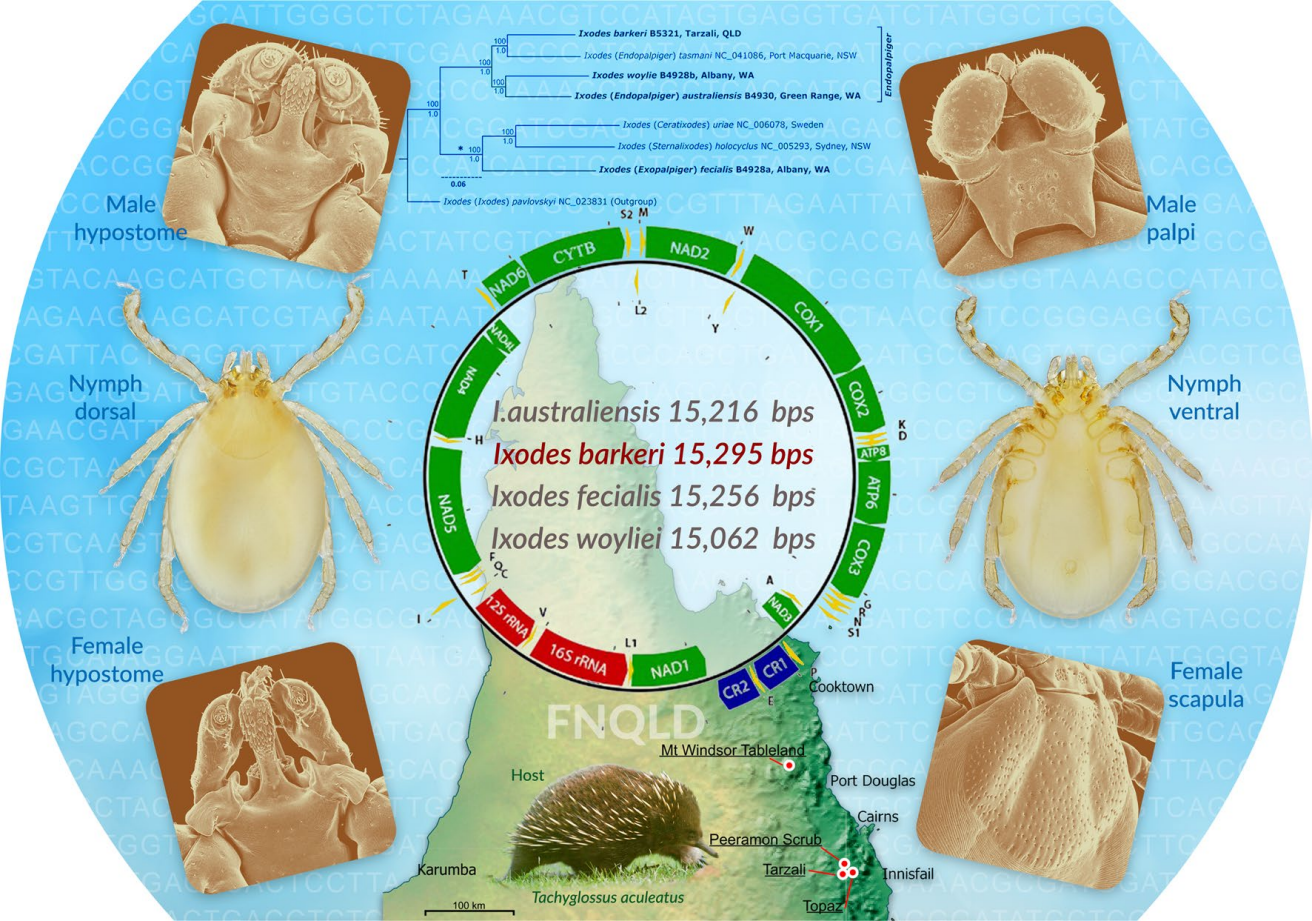

## Background

**Fig. 1:**
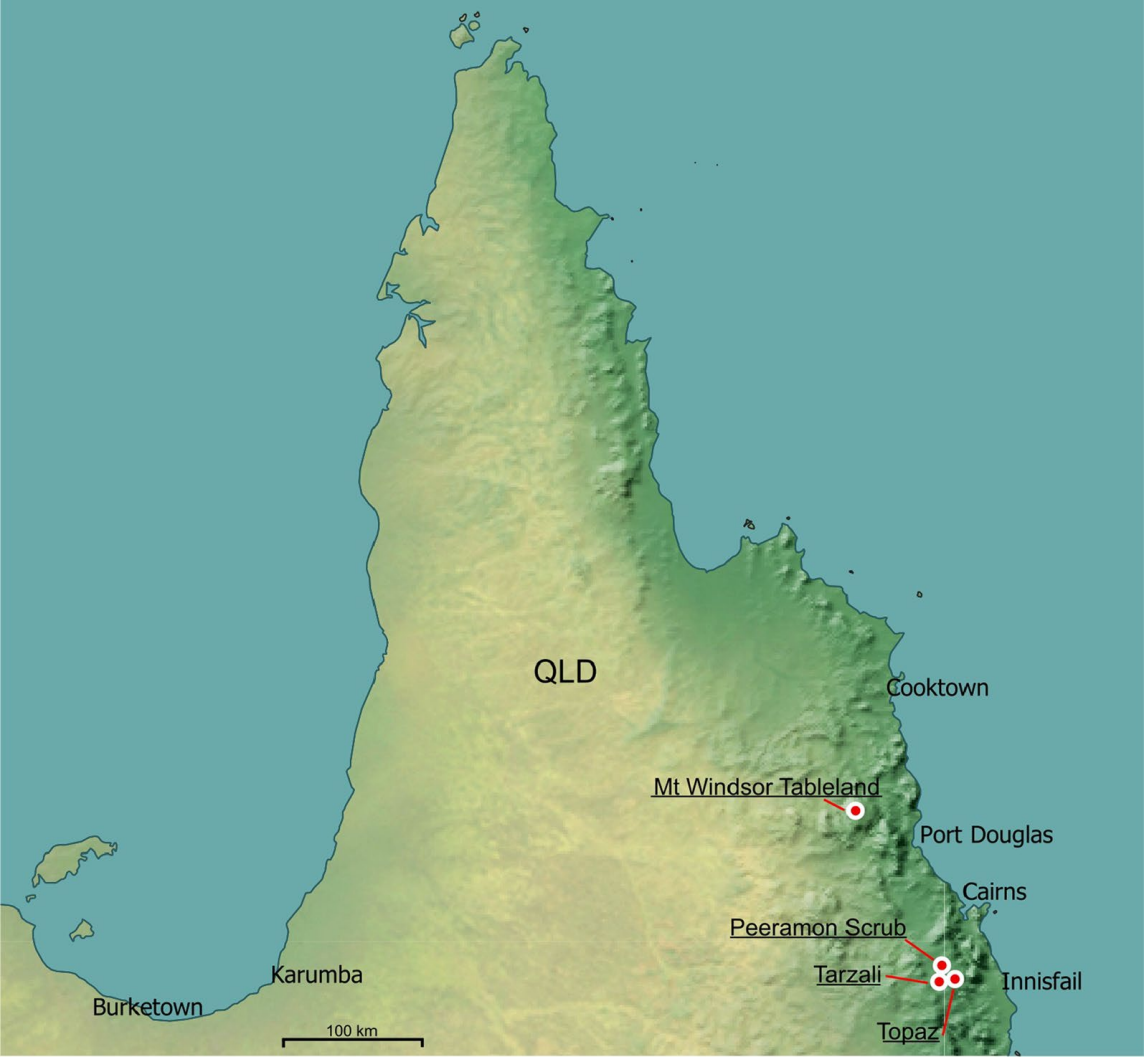
The four known localities in Australia, Queensland (Qld), of *Ixodes barkeri* Barker, 2019, are indicated by white-with-red dots

*Ixodes barkeri* Barker, 2019, was described from two male ticks collected from the short-beaked echidna *Tachyglossus aculeatus* (Shaw, 1792). One of these ticks was from rainforest of the Peeramon Scrub, Atherton Tableland, Far North Queensland (FNQ), whereas the other male was from an unknown locality in the vicinity of the Atherton Tableland. *Ixodes barkeri* is distinctive among Australian ticks, especially for its ventrolateral palpal projection, which is found only in an echidna tick from Papua New Guinea, *I. zaglossi* Kohls, 1960. *Ixodes zaglossi*, however, has syncoxae, whereas *I. barkeri* does not, and the ventrolateral palpal projection in *I. barkeri* is much bigger [1]. However, this extraordinary species, *I. barkeri*, could not be placed within a higher taxonomic framework, such as the subgenus classification of *Ixodes*, largely because of the lack of females. Since [1], we have acquired other specimens of *I. barkeri*: 6 males, 5 females, 34 nymphs and 2 larvae (Table 1, Fig. 1), allowing us to describe the female, nymph and larvae for the first time and to redescribe the male in greater detail to compare and contrast the morphology of *I. barkeri* with other species of the Australasian *Ixodes* clade (sensu [2]). We have also described the mitochondrial genome of *I. barkeri,* enabling inferences of phylogenetic relationships of this species with others in the genus.

**Table 1:** Specimens studied (morphology and/or genetics) in the present study. ANIC, Australian National Insect Collection; FMNH, Field Museum of Natural History (Chicago, IL)

Species	Host	Life stage/s	Place name	State	Date collected	Latitude	Longitude	Altitude (m)	Postcode	Collection no.	Collector	Collection	Lab—DNA preparation	Genbank no.
*I. australiensis*	*Macropus fuliginosus* (western grey kangaroo)	1F	Green Range	WA	16.10.2015	− 34.709878	118.413434	77	5153	B4930	SCB & DB	B&B	Nakao	OL597990
*I. fecialis*	*Isoodon obesulus* (southern brown bandicoot)	1F	Albany	WA	20.10.2015	− 35.051832	117.860732	ca. 10 m	6330	B4928a	Anne− Marie Horwitz & David Forshaw	B&B	Nakao	OL597993
*I. barkeri*	*Tachyglossus aculeatus* (short-beaked echidna)	1F	Tarzali	Qld	10.7.2017	− 17.43392	145.59726	768	4885	B5321-tube 1	A. Shima	B&B	NA	NA
*I. barkeri*	*Tachyglossus aculeatus* (short-beaked echidna)	3 M 3F	Tarzali	Qld	2016	− 17.43392	145.59726	768	4885	B5321-tube 3	A. Shima	B&B	NA	NA
*I. barkeri*	*Tachyglossus aculeatus* (short-beaked echidna)	34 N 2L	Tarzali	Qld	2016	− 17.43392	145.59726	768	4885	B5321-tube 2	A. Shima	B&B	Shao	OL597991
*I. barkeri*	*Tachyglossus aculeatus* (short-beaked echidna)	1 M	Topaz	Qld	2.7.2018	− 17.41452	145.71053	697	4885	B5322	A. Shima	B&B	Shao	OM302450
*I. barkeri*	Vegetation	1 M (holotype)	Peeramon Scrub	Qld	9.12.1995	− 17.31667	145.61667	750	4885	B4994/QM #MS 109845	G. Monteith	QM	NA	NA
*I. barkeri*	Vegetation	1F	Mt Windsor Tableland	Qld	unknown	− 16.21271	144.99863	1,187	4871	B6736	unknown	FMNH	NA	NA
*I. barkeri*	*Tachyglossus aculeatus* (short-beaked echidna)	1 M (paratype)	FNQ ("Cairns")(possibly Atherton Tableland)	Qld	15.05.2013	− 16.920348	145.7709529	3	4870	B3421/ANIC 48005261	I. Beveridge	ANIC	NA	NA
*I. woylie*	*Isoodon obesulus* (southern brown bandicoot)	1F	Albany	WA	20.10.2015 (confirmed from label in vial by RN)	− 35.051832	117.860732	ca. 10 m	6330	B4928b	Anne-Marie Horwitz & David Forshaw	B&B	Nakao	OL597992

The subgenera of *Ixodes* are morphologically ambiguous and in need of further refinement and testing with genetic data. In this regard, mitochondrial (mt) genomes have been remarkably instructive about the evolutionary history (phylogeny) of ticks (e.g. [3–7]). Thus, we sequenced the mt genomes of *I. barkeri* and its possible relatives that were available to us (*I. australiensis* Neumann, 1904, *I. fecialis* Warburton & Nuttall, 1909, and *I*. *woyliei* Ash et al*.* 2017) with a view to discovering which if any of the subgenera of *Ixodes* would be most suitable for *I. barkeri* Barker, 2019; [8] and [1] were not able to place *I. woyliei* and *I. barkeri* in a subgenus, respectively.

## Methods

### Material examined

Only field-collected ticks were available for study. The specimens were from the Barker and Barker Collection at the University of Queensland (Qld), the Queensland Museum (QM), the Australian National Insect Collection (ANIC) and the Field Museum of Natural History, Chicago, Illinois, USA (FM) (Table 1).

### Microscopy methods

Ticks were studied using a stereoscopic microscope (Nikon SMZ800N, Nikon Corp., Tokyo, Japan, and Olympus SZX16, Olympus Corp., Tokyo, Japan), compound microscope (Olympus BX53, Olympus Corp., Tokyo, Japan) and scanning electron microscope (JEOL JSM6610LV, JEOL Ltd., Tokyo, Japan). An ocular micrometre was used to measure ticks. Measurements are in millimetres for the adults, micrometres for the juveniles, and are given as the range followed by the mean and the number of specimens measured (n) in parentheses. Colour digital images were taken with a Canon 6D camera (Canon Corp., Tokyo, Japan).

Adobe Photoshop® software was used to correct images for broken legs and other damaged parts of the tick and to polish the image.

### Sequencing and assembly of mitochondrial genomes

Mitochondrial genomes were sequenced and assembled in two ways. First, the mt genome of *I. barkeri* Barker, 2019, was sequenced at Novogene Singapore and then assembled at the University of Queensland (UQ) by the protocol of [5]. DNA was prepared by us at the University of the Sunshine Coast and the University of Queensland. We extracted DNA from individual ticks and from various pools (groups of up to 3 ticks) of females, males, nymphs and larvae in an effort get adequate DNA for our experiments from all life-stages i.e. females, males, nymphs and larvae (Below we report that adequate DNA was obtained from a male and a pool of 3 nymphs but not from females nor larvae since the females and larvae had not been preserved well). Ticks were cut in half and then incubated at 56 °C for 62 h with Proteinase K to lyse the cells. The QIAGEN DNeasy Blood and Tissue kit was used to extract genomic DNA. The amount of DNA recovered was measured with Nanodrop and Qbit instruments. Groups of ticks that yielded > 200 nanograms (ng) of DNA were sent to Novogene Singapore for de novo library construction and next-generation Illumina sequencing. Groups of ticks with < 200 ng were combined with DNA from a different organism, usually a bird, to reach the minimum threshold of 200 ng of DNA required by Novogene Singapore. At Novogene Singapore, DNA was sonicated to fragment the DNA, and then fragments were end-polished, A-tailed and ligated with Illumina adaptors. DNA fragments were amplified with PCR, using P5 and P7 oligos, to create genomic libraries, which were purified with AMPure XP system. The Illumina Novaseq 6000 sequencing platform was used to generate two giga-bases of nucleotide sequence data (PE 150). We then constructed de novo contig assemblies of Illumina sequences in Geneious Prime [9] by the default assembler of Geneious Prime. Blast-searches of contigs revealed mt genes of ticks; these gene sequences were then assembled until entire mt genomes had been assembled.

Second, the mt genomes of *I. australiensis, I. fecialis* and *I. woylie* were sequenced at the Hokkaido University, Japan, and then assembled at the University of Queensland by the protocol of [5]. DNA was extracted from ticks with the NucleoSpin® DNA Insect (Macherey-Nagel, Germany). Entire mt genomes were then amplified in two overlapping fragments (long-range and short PCRs). Long-range PCR was used to amplify fragments that comprised about 12–13 kb of the mitogenome with the universal primers: mtG_K23 (5’-TCCTACATGATCTGAGTTYAGACCG-3’) and mtG_K25 (5’-AAAATTCWTAGGGTCTTCTTGTCC-3’) or mtG_K26 (5’-ACGGGCGATATGTRCATATTTTAGAGC-3’). Short PCRs were then used to amplify 1.5–2.5 kb of mt genomes with genus-specific primers. PrimeSTAR® GXL DNA Polymerase (Takara-Bio, Shiga, Japan) was used to amplify the long mt PCR products, whereas Tks Gflex™ DNA Polymerase (Takara-Bio) was used to amplify short mt gene fragments as well as nuclear rRNA genes. PCR conditions for PrimeSTAR® GXL DNA Polymerase were: 45 cycles of 98 °C for 10 s, 60 °C for 15 s and 68 °C for 10 min. PCR conditions for Tks Gflex™ DNA Polymerase were: 94 °C for 60 s, 45 cycles of 98 °C for 10 s, 55 °C for 15 s, 68 °C for 60 s and a final extension of 68 °C for 5 min. PCR products were examined on 1.2% agarose gels stained with Gel-Red™ (Biotium, Hayward, CA). PCR products were purified with a NucleoSpin Gel and PCR Clean Up Kit (Takara-Bio). Illumina sequencing libraries were constructed from the PCR fragments from the long-range and short PCR reactions with the Nextera DNA Library Prep Kit (Illumina, Hayward, CA) and were sequenced with the Illumina MiSeq platform with the MiSeq reagent kit v3 for 600 cycles.

### Annotation of mitochondrial genomes

Mitochondrial genomes were annotated with Geneious Prime. Protein-coding genes were identified by searches with BLAST [10] for open reading frames. Regions between protein-coding genes were searched with BLAST [10] to find rRNA genes, tRNA genes and control regions. The tRNA that we expected to find but did not find with BLAST was found with the aid of the tRNAscan-SE Search Server v1.21 [11] and the MITOS Web Server [12]. The nucleotide sequences of tRNA genes were confirmed by studying the putative secondary structure of transcripts, as implemented in Geneious Prime [9].

### Phylogenetic methods

Phylogenies were inferred by both maximum likelihood (ML) and Bayesian inference (BI) methods implemented in the RAXML-HPC2 v 8.2.12 [13] and MrBayes v3.2.2 [14], respectively. JmodelTest2 v2.1.6 [15] was used to find the optimal substitution model for the nucleotide dataset. The GTR + G + I model was found to be the best fit for our dataset. In all ML and BI runs (experiments), genes were partitioned. Rapid bootstrapping of 1000 replicates of our data was executed in RAXML-HPC2 v 8.2.12 [13]. There were two simultaneous BI runs: 10 million generations sampled every 1000 MCMC steps. For every BI run, four MCMC chains (three heated and one cold) were executed. The first 25% of steps was discarded as burn-in. Tracer v 1.5 [16] was used to observe the effective sample size (ESS) and convergence of independent runs. Phylogenetic trees were displayed in FigTree v 1.4.4 [17]. Branch support in the phylogenetic trees generated by RAXML-HPC2 v 8.2.12 [12] and MrBayes v 3.2.2 [14] was assessed by the bootstrap values and posterior probability values, respectively. All phylogenies were inferred through the CIPRES Science Gateway v.3.3 [18]. *Ixodes pavlovskyi* Pomerantzev, 1946, a species from the clade of the “other *Ixodes*” (sensu [2]) was the out-group.

## Results

### Systematics

**Family Ixodidae Murray**, 1877

**Genus *Ixodes* Latreille**, 1795

**Subgenus *Endopalpiger* Schulze**, 1935

Figures 2, 3, 4, 5, 6, 7, 8, 9, 10

**Fig. 2:**
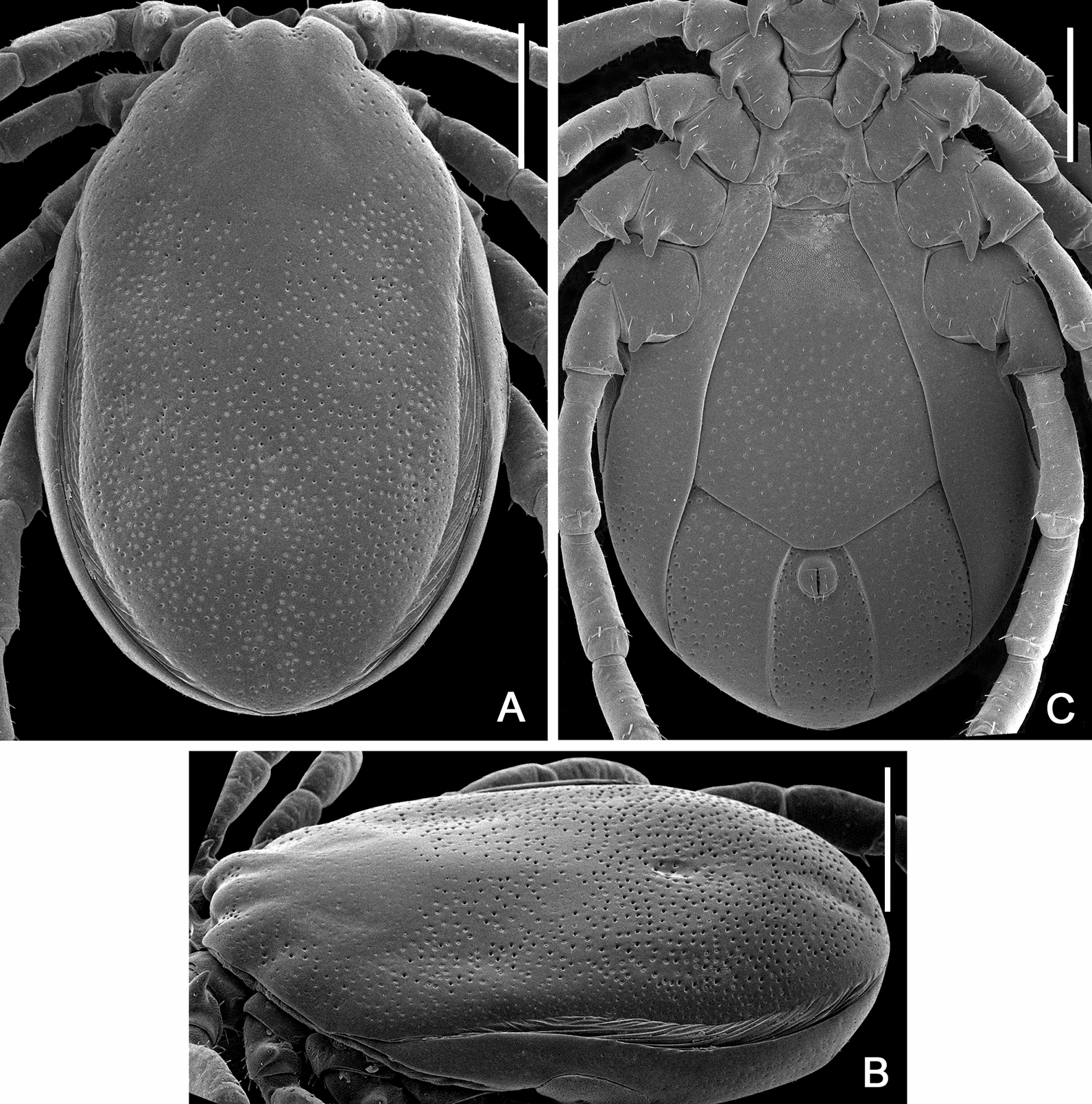
*Ixodes barkeri* Barker, 2019, scanning electron micrographs of idiosoma of male. **A** Dorsal view; **B** dorsolateral view; **C** ventral view. *Scale bars*: 0.5 mm

***Ixodes barkeri*** **Barker, 2019**

**Fig. 3:**
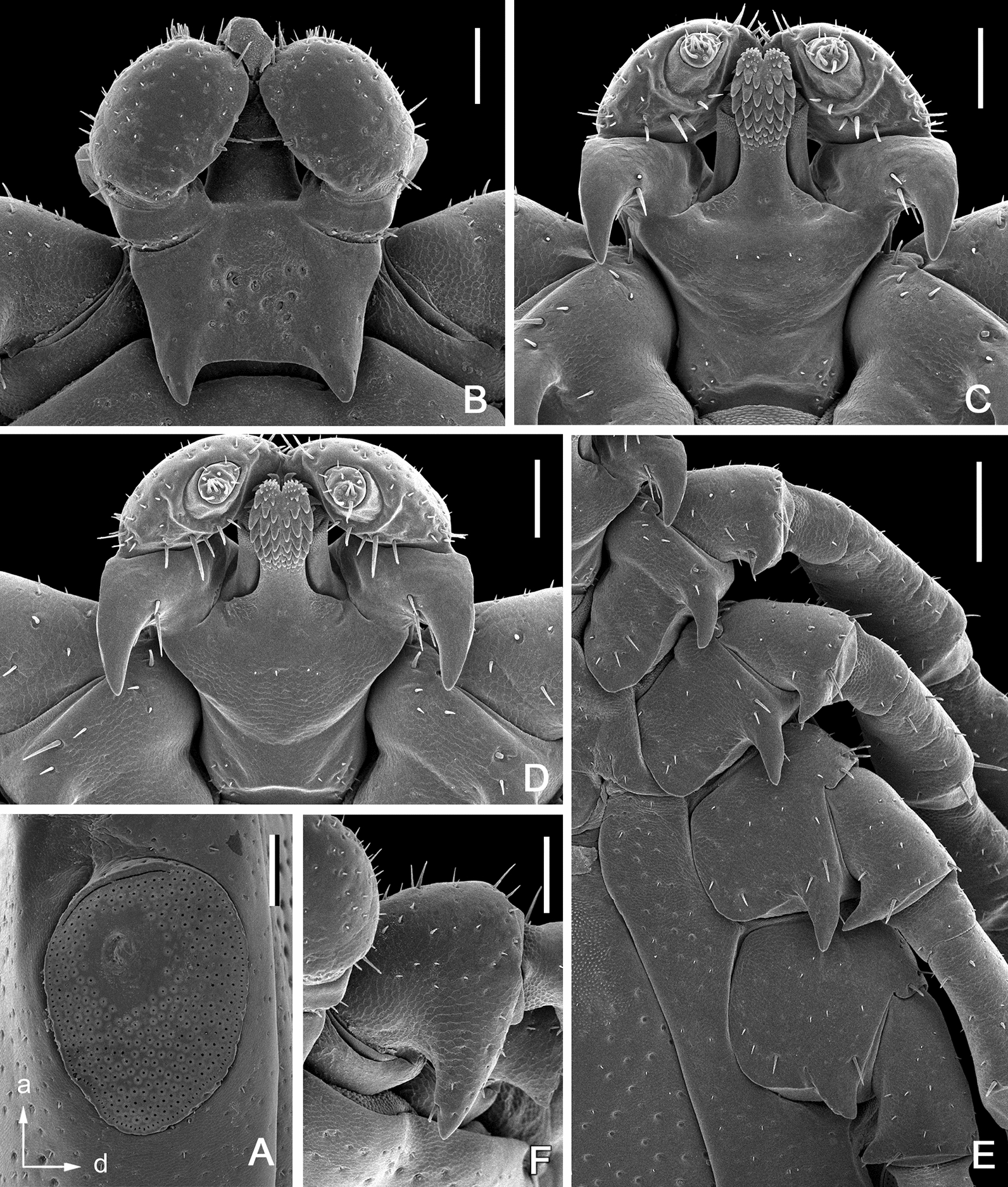
*Ixodes barkeri* Barker, 2019, scanning electron micrographs of male. **A** Spiracular plate (*arrows* show orientation of spiracular plate: a, anterior; d, dorsal). **B** Gnathosoma, dorsal view. **C** Gnathosoma, ventral view. **D** Gnathosoma, anteroventral view. **E** Coxae. **F** Trochanter I, dorsal view. *Scale bars*: **A**–**D**, **F** 0.1 mm; E, 0.2 mm

**Male***.* (Based on 6 specimens, 3 measured—refer to Table 1; Figs. 2, 3, 8) Idiosoma (Fig. 2) broadly elongate-oval with broadly rounded posterior margin, widest slightly posterior to mid-length; length from apices of scapulae to posterior body margin 2.17–2.59 mm (2.42 mm), width 1.53–1.72 (1.61), ratio 1.42–1.56 (1.49). Lateral groove distinct. Conscutum (Figs. 2, 8) length 2.12–2.56 (2.38), width 1.37–1.48 (1.44), ratio 1.54–1.73 (1.65); laterally and postero-laterally with a distinct narrow non-sclerotised band; scapulae short, blunt; lateral carinae absent; cervical grooves indistinct; dense moderately large punctations evenly distributed over conscutum, except for pseudoscutum area; pseudoscutum with indistinct punctations; setae moderately dense, very short (*c.* 0.01) and indistinct (Fig. 2). Venter plate outlines as illustrated (Fig. 2); median plate: length 1.06–1.18 (1.10), width 0.74–0.84 (0.81), ratio 1.26–1.43 (1.37); adanal plate: length 0.74–0.82 (0.79), width 0.42–0.48 (0.45), ratio 1.67–1.95 (1.77); anal plate: 0.54–0.60 (0.58), width 0.32–0.36 (0.34), ratio 1.67–1.71 (1.69). All ventral plates with dense, moderately large punctations (Figs. 1, 2). Genital aperture (Fig. 2C) located at level of posterior margin of coxae II; posterior margin of genital apron deeply concave. Ventral setae (Fig. 2) moderately dense, very short, evenly distributed on all plates; length of setae on median plate *c.* 0.01. Anal groove (Fig. 2C) straight anteriorly and open posteriorly. Spiracular plate (Fig. 2A) broadly oval, longer than wide, length 0.34–0.42 (0.38), width 0.28–0.34 (0.31), ratio 1.21–1.25 (1.23).

Gnathosoma (Figs. 3, 8) length from palpal apices to cornual apices dorsally 0.45–0.48 (0.46), width between lateral projection of palpal segments I 0.43–0.48 (0.46), ratio 0.98–1.04 (1.01). Dorsal basis capituli (Fig. 3A) length from medial insertion of palpal segment I to cornual apices 0.21–0.25 (0.23), width 0.32–0.35 (0.33), ratio width to length 1.41–1.52 (1.46), subrectangular, posterior margin nearly straight; cornua long, ratio total length of basis capituli, including cornua, to cornual length 4.83–5.78 (5.39), triangular with narrowly rounded apex. Ventral basis capituli (Fig. 3C) subrectangular; lateral margins with slight constrictions at mid-length; auriculae absent; short converging ridges in auricular areas. Palpi (Fig. 3B) short, length dorsally (segments II and III) 0.23–0.24 (0.23), maximum width (in dorsolateral plane) 0.18–0.19 (0.19), ratio 1.22–1.29 (1.26), length of palpal segment I ventrally 0.16 (*n* = 1), maximum width ventrally 0.17 (*n* = 1), ratio 0.94 (*n* = 1); segment I greatly enlarged, greatest dimension in anteromedian-posterolateral direction; dorsally segment I subrectangular; ventrally segment I subtriangular, posterior margin with very long and narrow spur with sharply pointed apex; segments II and III fused together with indistinct suture between them, narrower proximally and abruptly widening to broadly rounded apex. Hypostome (Fig. 3C) length 0.18–0.19 (0.18), width 0.08–0.10 (0.09), ratio 1.75–2.10 (1.95); club-shaped, widening to broadly rounded apex with medial indentation; base of hypostome at level of base of palpal segment I; dental formula 3/3, basal half of hypostome without denticles, denticles sharply pointed.

Legs moderately long, slender. Coxae (Figs. 2E, 9): coxae I–IV with long and narrow external spur with narrowly rounded to sharply pointed apex; spur on coxae I–III subequal, spur on coxa IV nearly twice shorter those on coxae I–III; coxae I–IV without syncoxae. Trochanter I with long, triangular spur with sharply pointed apex; trochanters I–IV with long, narrow, with sharply pointed apex spur ventrally. Tarsus I: length 0.56–0.60 (0.58); tarsus IV length 0.53–0.58 (0.55); tarsi only slightly humped subapically.

**Fig. 4:**
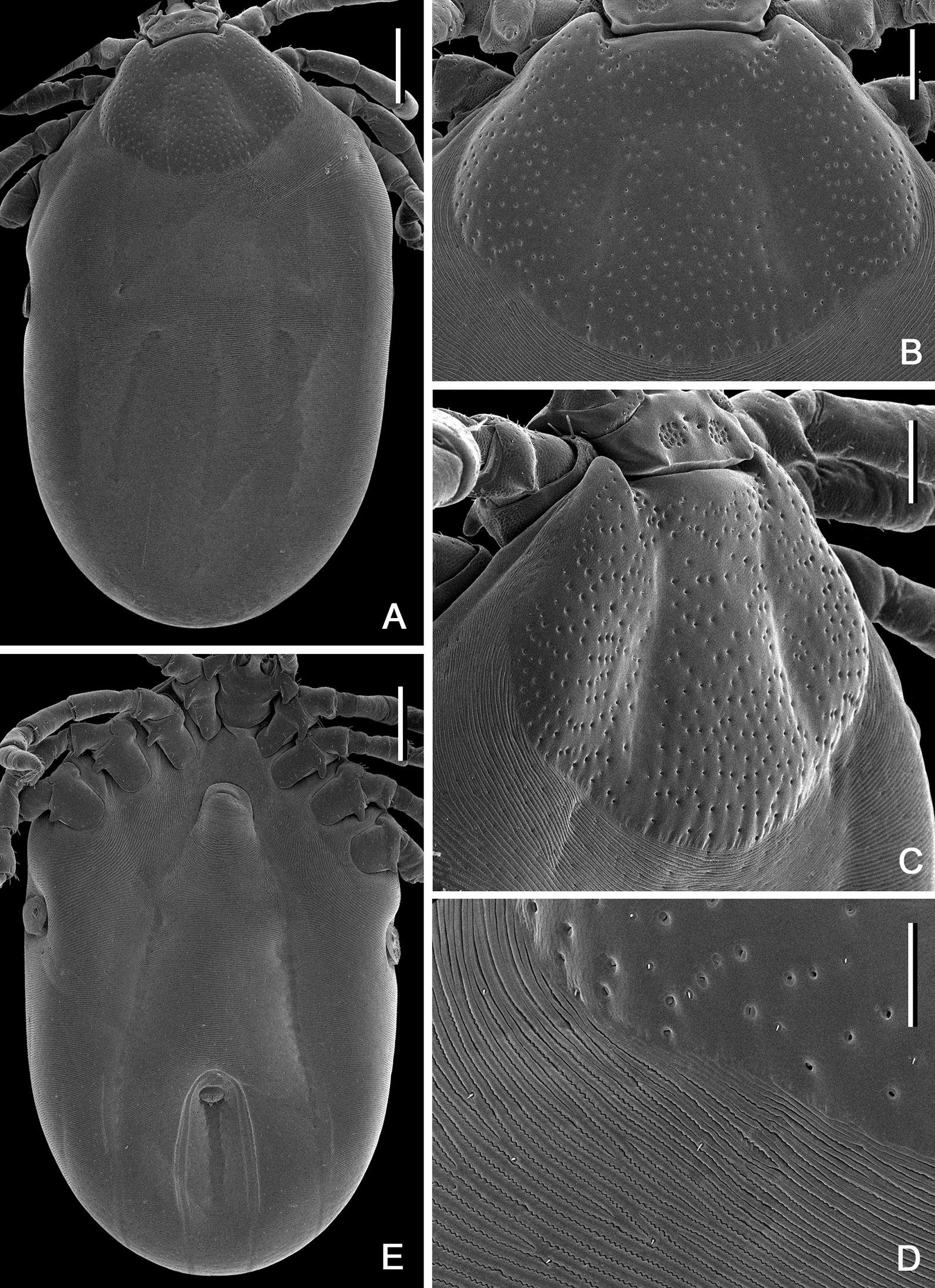
*Ixodes barkeri* Barker, 2019, scanning electron micrographs of female. **A** Idiosoma, dorsal view. **B** Scutum, dorsal view. **C** Scutum, dorsolateral view. **D** Idiosoma showing scutum and alloscutum with punctations and setae, dorsal centrolateral portion. **E** Idiosoma, ventral view. *Scale bars*: **A**, **E** 0.5 mm; **B**, **C** 0.2 mm; **D** 0.1 mm

**Fig. 5:**
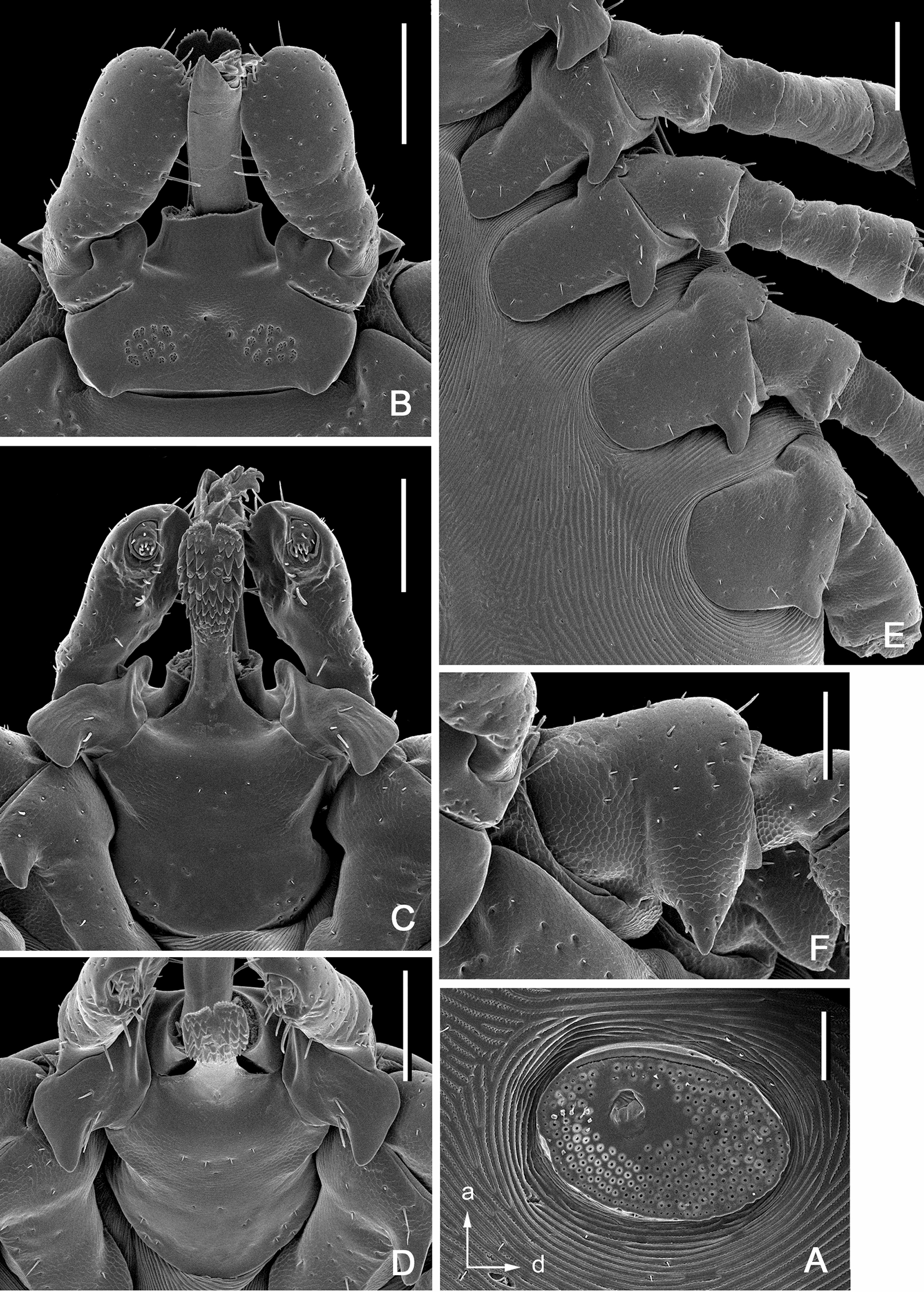
*Ixodes barkeri* Barker, 2019, scanning electron micrographs of female. **A** Spiracular plate (*arrows* show orientation of spiracular plate: a, anterior; d, dorsal). **B** Gnathosoma, dorsal view. **C** Gnathosoma, ventral view (**I**, palpal article 1; **II**, palpal article 2; **ss** the strongly salient part of palpal article 1). **D** Gnathosoma, anteroventral view. **E** Coxae. **F** Trochanter **I**, dorsal view. *Scale bars*: A, F, 0.1 mm; B–E, 0.2 mm

**Female.** (Based on 1 to 5 specimens—refer to Table 1; Figs. 4, 5, 9) Idiosoma (Figs. 4A, 9) length from scapular apices to posterior body margin in moderately engorged specimen 4.1 (*n* = 1), width in moderately engorged 2.5 (*n* = 1), ratio 0.16 (*n* = 1), broadly suboval, widest approximately at mid-length. Scutum (Figs. 4, 9) length 1.05–1.18 (1.13; *n* = 4), width 1.40–1.58 (1.49; *n* = 4), ratio 0.73–0.79 (0.75; *n* = 4); lateral margins diverging for approximately 2/3 of scutum length, broadly rounded posteriorly; lateral carinae lacking; cervical grooves shallow; dense, small punctations evenly distributed throughout scutum; setae (Fig. 4) relatively sparse, very short (*c.* 0.005), indistinct and nearly equal to those on alloscutum, distributed as figured. Alloscutum (Fig. 4) as illustrated; setae of alloscutum (Fig. 4C) numerous, evenly distributed, very short, length of setae in central field *c.* 0.01, indistinct. Venter (Fig. 4E) as illustrated; genital aperture (Fig. 4E) medial to coxae III; genital groove (Fig. 4E) well developed; anal groove (Fig. 4E) oval with open posterior margin; ventral setae numerous, length of preanal setae *c.* 0.01, evenly distributed. Spiracular plates (Fig. 5A) length 0.27–0.32 (0.30; *n* = 4), width 0.39–0.47 (0.44; *n* = 4), ratio 0.68–0.70 (0.69; *n* = 4); broadly oval; marginal row of perforations in groove anteriorly.

Gnathosoma (Fig. 5B) length from palpal apices to cornual apices dorsally 0.60–0.70 (0.65; *n* = 4), width between lateral projection of palpal segments I 0.66–0.74 (0.71; *n* = 4), ratio 0.89–0.95 (0.92; *n* = 4). Dorsal basis capituli (Fig. 5B) length from medial insertion of palpal segment I to cornual apices 0.22–0.25 (0.24; *n* = 4), width 0.51–0.55 (0.54; *n* = 4), ratio width to length 2.20–2.32 (2.26; n = 4), subrectangular, posterior margin nearly straight; cornua short, ratio of total length of basis capituli (including cornua) to cornual length 17.50–20.00 (19.12; *n* = 4), triangular with broadly rounded apex; subcircular porose areas poorly distinct, not indented, separated by distance nearly equal to their own width, several openings arranged in discrete punctations. Ventral basis capituli (Fig. 5C) subrectangular; lateral margins with slight constrictions at mid-length; auriculae absent; short converging ridges in auricular areas. Palpi (Fig. 5B) short, length dorsally (segments II and III) 0.41–0.50 (0.46; *n* = 4), maximum width (in dorsolateral plane) 0.21–0.25 (0.23; *n* = 4), ratio 1.85–2.10 (1.95; n = 4), length of palpal segment I ventrally 0.31–0.36 (0.34; *n* = 4), maximum width ventrally 0.19–0.20 (0.20; *n* = 4), ratio 1.64–1.75 (1.68; n = 4); segment I greatly enlarged, greatest dimension in anteromedian-posterolateral direction; dorsally segment I subrectangular with convex medial margin; ventrally segment I subtriangular, posterior margin with long moderately narrow spur with narrowly rounded apex; segments II and III fused together with indistinct suture between them, narrower proximally and abruptly widening to broadly rounded apex. Hypostome (Fig. 5C) length 0.36–0.40 (0.38; *n* = 4), width 0.12–0.16 (0.14; *n* = 4), ratio 2.46–2.85 (2.68; *n* = 4); club-shaped, widening to broadly rounded apex with medial indentation; base of hypostome approximately at level of base of palpal segment II; dental formula 4/4 (few rows may be 3/3), basal half of hypostome without denticles, denticles sharply pointed.

Legs moderately long, slender. Coxae (Figs. 5E, 9): coxae I–IV with moderately long and narrow external spur with narrowly rounded apex; spur on coxae I–III subequal, spur on coxa IV nearly twice shorter than those on coxae I–III; coxae I–IV without syncoxae. Trochanter I with moderately long, triangular spur with sharply pointed apex; trochanters I–IV without spur ventrally. Tarsus I: length 0.66–0.76 (0.73; *n* = 4); tarsus IV length 0.57–0.66 (0.63; *n* = 4); tarsi only slightly humped subapically.

**Fig. 6:**
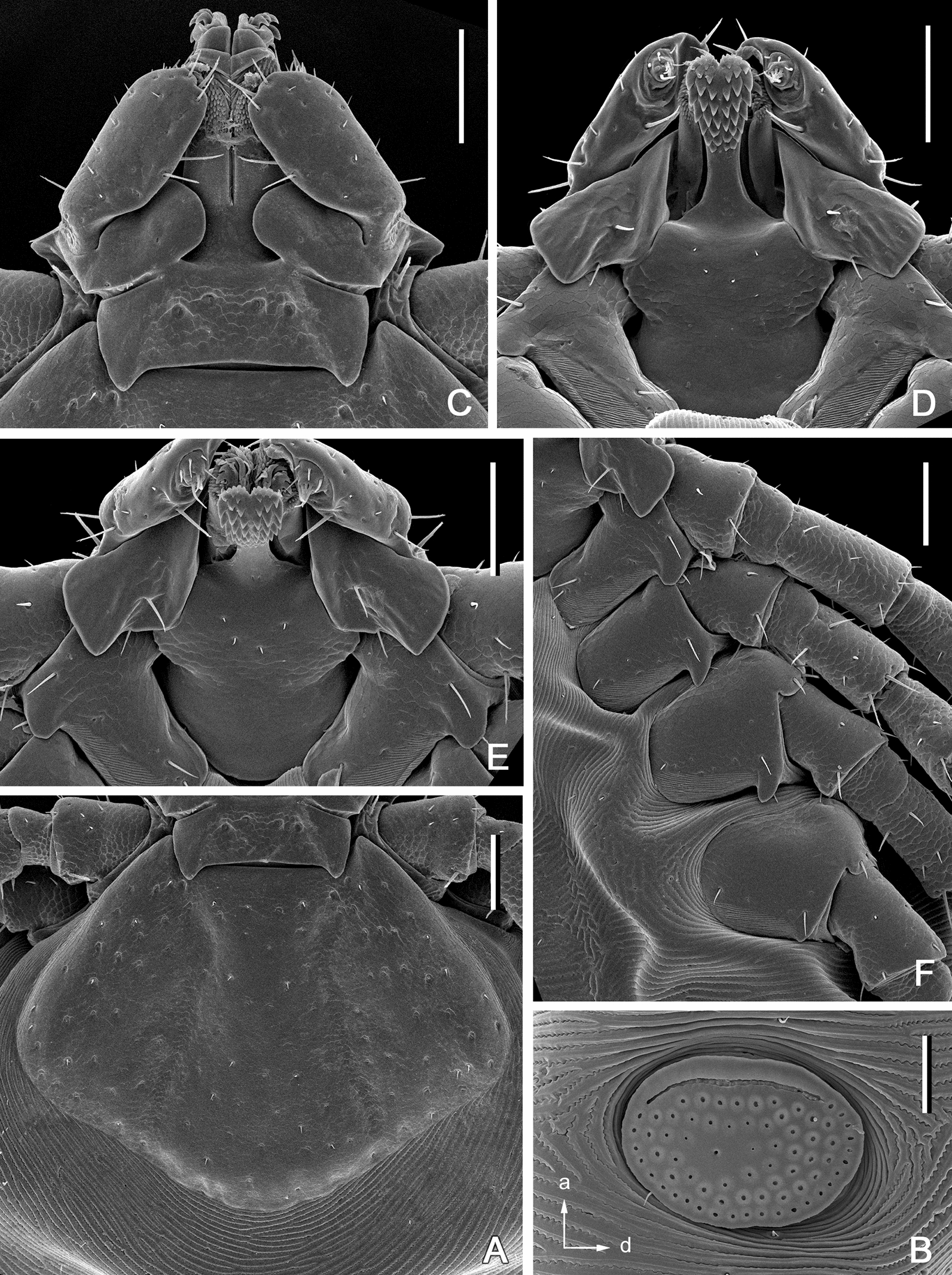
*Ixodes barkeri* Barker, 2019, scanning electron micrographs of nymph. **A** Scutum. **B** Spiracular plate (*arrows* show orientation of spiracular plate: a, anterior; d, dorsal). **C** Gnathosoma, dorsal view. **D** Gnathosoma, ventral view. **E** Gnathosoma, anteroventral view. **F** Coxae. *Scale bars*: A, C–F, 0.1 mm; B, 0.05 mm

**Nymph.** (Based on 34 specimens—refer to Table; Fig. 6) Scutum (Fig. 6A) length 515–520 (518; *n* = 2), width 690–700 (695; *n* = 2), ratio 0.74–0.75 (0.75; *n* = 2); lateral margins diverging for approximately half of scutum length, broadly rounded posteriorly; posterolateral margin with slight indentations; lateral carinae lacking; cervical grooves shallow; moderately dense, small punctations evenly distributed throughout scutum; setae (Fig. 6A) relatively sparse, very short: length in central field of scutum 10 (*n* = 2), indistinct and nearly equal to those on alloscutum, distributed as figured. Setae of alloscutum numerous, evenly distributed, very short, length of setae in central field 10–13 (11; *n* = 2), indistinct. Anal groove oval with open posterior margin; ventral setae numerous, evenly distributed. Spiracular plates (Fig. 6B) broadly oval; marginal row of perforations in groove anteriorly.

Gnathosoma (Fig. 6C) length from palpal apices to cornual apices dorsally 285 (*n* = 2), width between lateral projection of palpal segments I 375–380 (378; *n* = 2), ratio 0.75–0.76 (0.76; *n* = 2). Dorsal basis capituli (Fig. 6C) length from medial insertion of palpal segment I to cornual apices 108 (*n* = 2), width 238–240 (239; *n* = 2), ratio width to length 2.21–2.23 (2.22; *n* = 2), subrectangular, posterior margin nearly straight; cornua moderately long, triangular with narrowly rounded apex. Ventral basis capituli (Fig. 6D) subrectangular; lateral margins with slight constrictions at mid-length; auriculae absent; short converging ridges in auricular areas. Palpi (Fig. 6C) short, length dorsally (segments II and III) 190–193 (191; *n* = 2), width 73–85 (79; *n* = 2), ratio 2.24–2.66 (2.45; *n* = 2), length of palpal segment I ventrally 140–150 (145; *n* = 2), maximum width ventrally 125–130 (128; *n* = 2), ratio 1.12–1.15 (1.14; *n* = 2); segment I greatly enlarged, greatest dimension in anteromedian-posterolateral direction; dorsally segment I subrectangular with convex medial margin; ventrally segment I subtriangular, posterior margin with long moderately broad spur with narrowly rounded apex; segments II and III fused together with indistinct suture between them, narrower proximally and abruptly widening to broadly rounded apex. Hypostome (Fig. 6D) length 150 (*n* = 1), width 73 (*n* = 1), ratio 2.07 (*n* = 1); club-shaped, widening to broadly rounded apex with medial indentation; base of hypostome approximately at level of base of palpal segment II; dental formula 3/3 (few basal rows 2/2), basal half of hypostome without denticles, denticles sharply pointed.

Legs moderately long, slender. Coxae (Fig. 6F): coxae I–IV with external spur; spur on coxae I–III moderately long, nearly subequal; spur on coxa I with broadly rounded apex, spur on coxae II and III with narrowly rounded to sharply pointed apex; spur on coxa IV very short; coxae I–IV with syncoxae occupying approximately 1/3, 1/4, 1/5 and 1/6 respectively of coxal width. Trochanters I–IV without spur ventrally. Tarsus I: length 320–335 (328; *n* = 2); tarsus IV length 298–310 (304; *n* = 2); tarsi only slightly humped subapically.

**Fig. 7:**
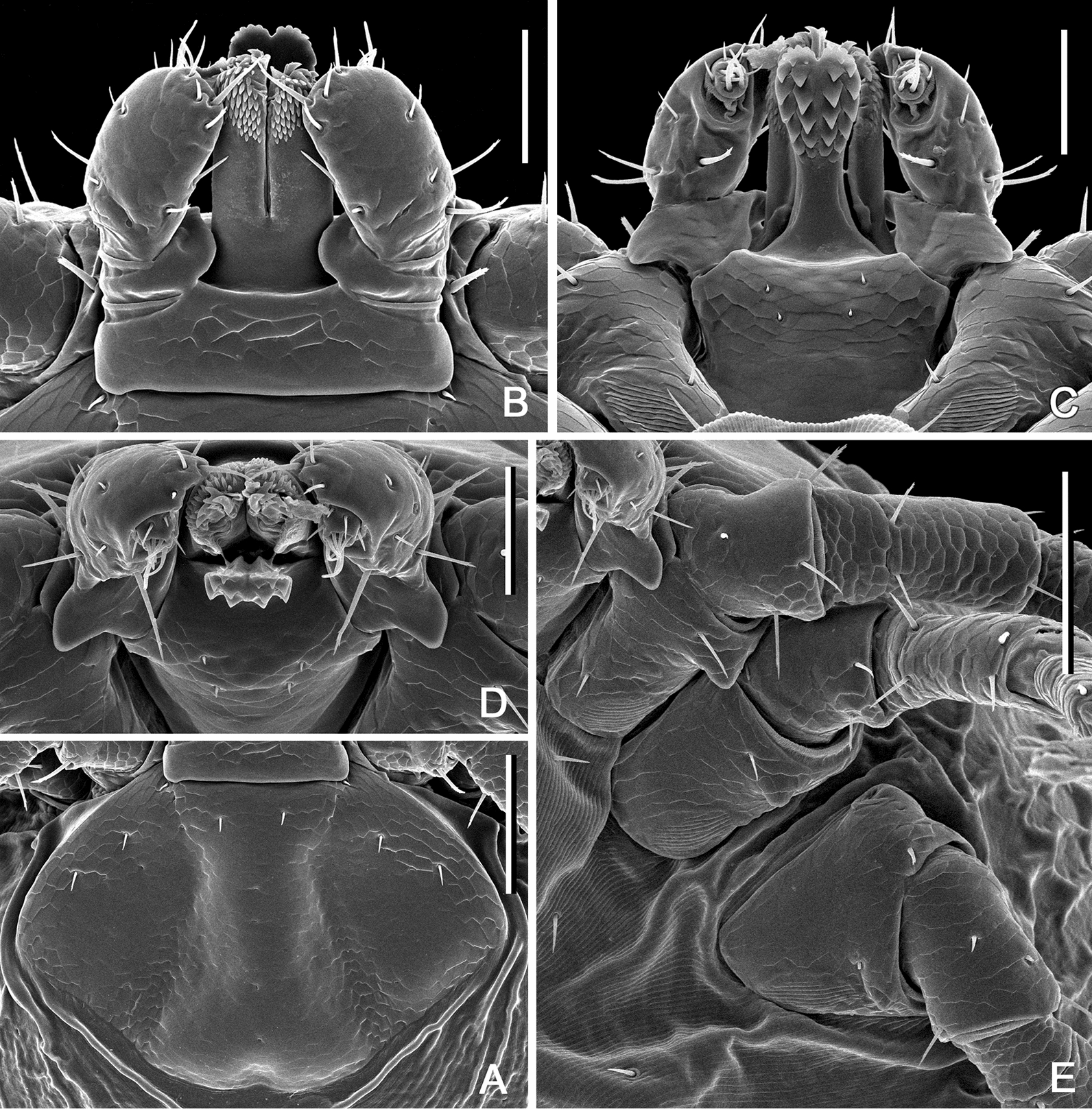
*Ixodes barkeri* Barker, 2019, scanning electron micrographs of larva. **A** Scutum. **B** Gnathosoma, dorsal view. **C** Gnathosoma, ventral view. **D** Gnathosoma, anteroventral view. **E** Coxae. *Scale bars*: **A**, **E** 0.1 mm; **B**–**D**, 0.05 mm

**Larva.** (Based on 2 specimens—refer to Table 1; Figs. 7, 10) Scutum (Fig. 7A) length 238 (*n* = 1), width 360 (*n* = 1), ratio 0.66 (*n* = 1); lateral margins diverging for approximately half of scutum length, broadly rounded posteriorly; posterolateral margin with slight indentations; lateral carinae lacking; cervical grooves shallow; setae 3 pairs, length of Sc_1_ 11 (*n* = 1); length of Sc_4_ 14 (*n* = 1). Dorsal setae of alloscutum undetermined in number since we only had larvae that were engorged to examine: it was impossible to confidently count and associate setae of the idiosoma dorsally and ventrally. Length of Cd_1_ 17 (*n* = 1), length of Md_1_ 23 (*n* = 1). Ventral setae undetermined number; 1 pair on anal valves; 3 pairs of sternals, length of St_1_ 19 (*n* = 1); 2 pairs of preanals, length of Pa_1_ 22 (*n* = 1), length of Pa_2_ 32 (*n* = 1).

**Fig. 8:**
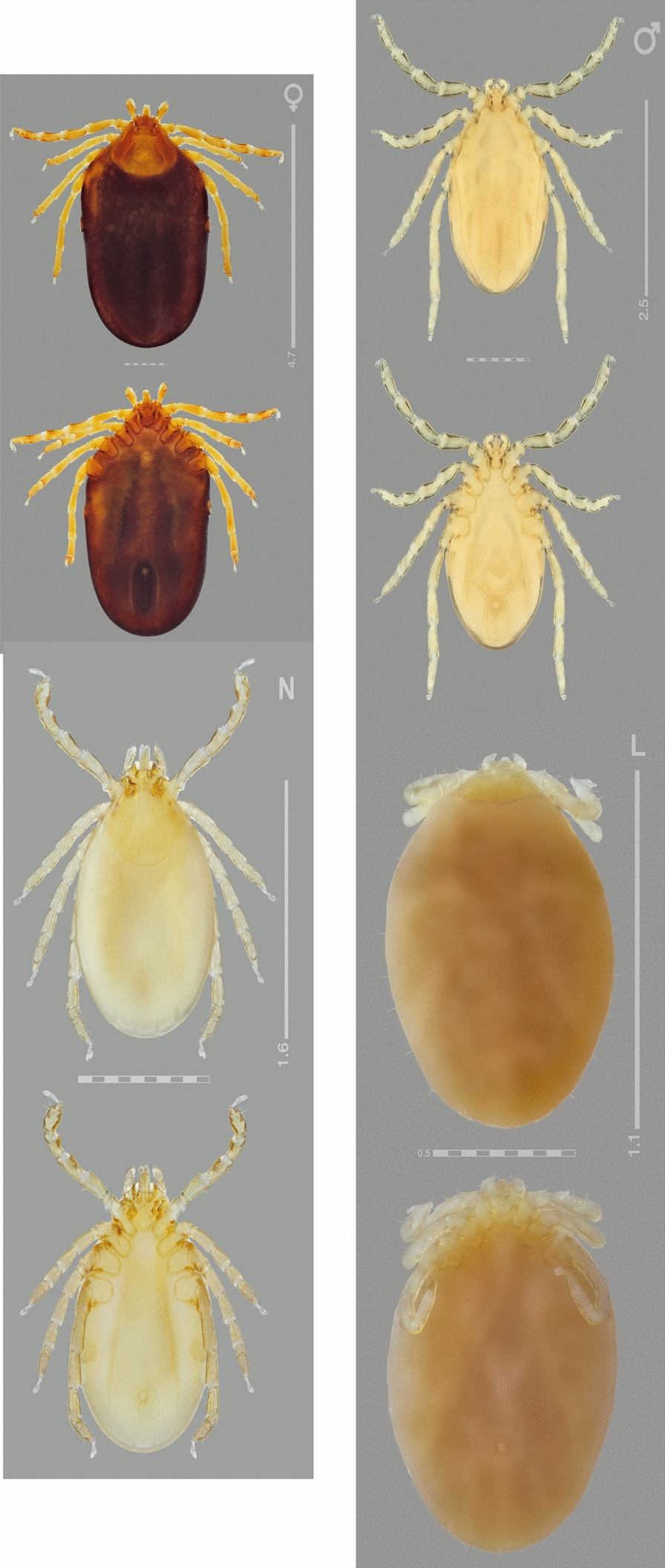
*Ixodes barkeri* Barker, 2019, light microscopy image of female (Barker & Barker Collection reference #B5321), male (# B4994), nymph (#B5321) and larva (# B5321). Horizontal broken scale bars: 1 mm; vertical scale bars also in mm

Gnathosoma (Fig. 7B–D) length from palpal apices to cornual apices dorsally 123 (*n* = 1), width between lateral projection of palpal segments I 155 (*n* = 1), ratio 0.79 (*n* = 1). Dorsal basis capituli (Fig. 7B) width 135 (*n* = 1), subrectangular, posterior margin nearly straight; cornua practically indistinct. Ventral basis capituli (Fig. 7C) subrectangular; lateral margins with slight constrictions at mid-length; auriculae absent. Post-hypostomal setae two pairs, length of Ph_1_ 4 (*n* = 1), length of Ph_2_ 4 (*n* = 1); distance between Ph_1_ 37 (*n* = 1), distance between Ph_2_ 27 (*n* = 1). Palpi (Fig. 7B) short, length dorsally (segments II and III) 77 (*n* = 1), maximum width (in dorsolateral plane) 42 (*n* = 1), ratio 1.83 (*n* = 1), maximum width of palpal segment I ventrally 50 (*n* = 1); segment I greatly enlarged, greatest dimension in anteromedian-posterolateral direction; dorsally segment I subrectangular with convex medial margin; ventrally segment I subtriangular, posterior margin with long moderately broad spur with narrowly rounded apex; segments II and III fused together with indistinct suture between them, narrower proximally and abruptly widening to broadly rounded apex; segment I lacking setae, segments II and III combined with nine dorsal and three ventral setae. Hypostome (Fig. 7C) length 83 (*n* = 1), width 34 (*n* = 1), ratio 2.44 (*n* = 1); club-shaped, widening to broadly rounded apex with medial indentation; base of hypostome approximately at level of mid-length of palpal segment I; dental formula 2/2, approximately five denticles in files; basal half of hypostome without denticles, denticles sharply pointed.

Legs moderately long, slender. Coxae (Fig. 7E): coxae I–III with external spur; spur on coxa I moderately long, on coxa II and III short to very short; spur on coxae I–III with broadly rounded apex; coxae I–III with syncoxae occupying approximately 1/3, 1/4 and 1/5 respectively posteromedian portion of coxal width. Trochanters I–IV without spur ventrally. Tarsus I: length 183 (*n* = 1); tarsus III length 166 (*n* = 1); tarsi only slightly humped subapically.

#### Remarks

By having a greatly enlarged palpal segment I that extends inwardly and anteriorly, all active life stages of *I. barkeri* most closely resemble those of the *Endopalpiger* species of Australasia: *I. acer*, *I. australiensis*, *I. giluwensis*, *I. hydromyidis*, *I. luxuriosus*, *I. mirzai*, *I. planiscutatus*, *I. steini*, *I. stellae*, *I. tasmani*, *I. victoriensis*, *I*. *woyliei* and *I. zaglossi* (refer to [8, 19, 20]).

The males of *I. australiensis*, *I. tasmani*, *I. victoriensis* and *I. zaglossi* have been described [19, 21–24]. The male of *I. barkeri* is easily distinguished from the males of the *Endopalpiger* species by the absence of the syncoxal areas on all coxae (vs. well-developed syncoxae on coxae I–IV in all those species).

The female of *I. barkeri* resembles only that of *I. woyliei* by the absence of syncoxal areas on coxae (vs. females of all other *Endopalpiger* with well-developed syncoxae). The female of *I. barkeri* can be differentiated from *I. woyliei* by the scutum and basis capituli dorsally and ventrally without lateral carinae and other longitudinal ridges (vs. lateral carinae and longitudinal ridges present in *I. woyliei*), the considerably smaller palpal segment I with a long spur on its posterior margin (vs. greatly enlarged palpal segment I with shorter spur on its posterior margin in *I. woyliei*), 4/4 dental formula on hypostome (vs. 6/6 in *I. woyliei*) and the long spur on trochanter I dorsally (vs. indistinct spur in *I. woyliei*).

The nymph of *I. australiensis*, *I. hydromyidis*, *I. luxuriosus* ([25] wrote that the nymph of *I. luxuriosus* had not been described, although there is a brief description of it in [26]), *I. steini*, *I. tasmani*, *I. victoriensis* and *I. woyliei* have been described [8, 19, 22, 24, 26]. Unfortunately, all of these published descriptions and illustrations are too brief for confident comparison. Nonetheless, we note that the nymph of *I. barkeri* has a scutum without lateral carinae (vs. distinct carinae in *I. victoriensis*), a scutum and basis capituli dorsally and ventrally without distinct longitudinal ridges (vs. with distinct, sharp ridges in *I. woyliei*), a distinct cornua (vs. no cornua in *I. australiensis*, *I. hydromyidis*, *I. tasmani* and *I. woyliei*), mostly 3/3 dental formula on the hypostome (vs. 2/2 dental formula in *I. hydromyidis* and *I. tasmani*, 4/4 in *I. australiensis*), external spurs on coxae I–IV (vs. apparently no spurs on coxae in *I. hydromyidis*, *I. luxuriosus*, *I. steini* and *I. tasmani*) and tarsi I–IV slightly humped subapically, without a notch (vs. strongly humped tarsi with distinct notch in *I. victoriensis*).

The larvae of *I. hydromyidis*, *I. tasmani* and *I. victoriensis* have been described [22, 24, 27, 28]. Unfortunately, as with the nymphs, all of these published descriptions and illustrations of larvae are too brief for confident comparison. Nonetheless, we note that the larva of *I. barkeri* has indistinct cornua on the basis capituli dorsally (vs. distinct cornua in *I. victoriensis*) and has external spurs on coxae I–III (vs. no spurs on coxae in *I. hydromyidis* and *I. tasmani*).

Our diagnoses may be broadened and improved once the nymphs and larvae of the other Australasian species of *Endopalpiger* are redescribed and illustrated accurately.

### Mitochondrial genomes and phylogeny

**Fig. 9:**
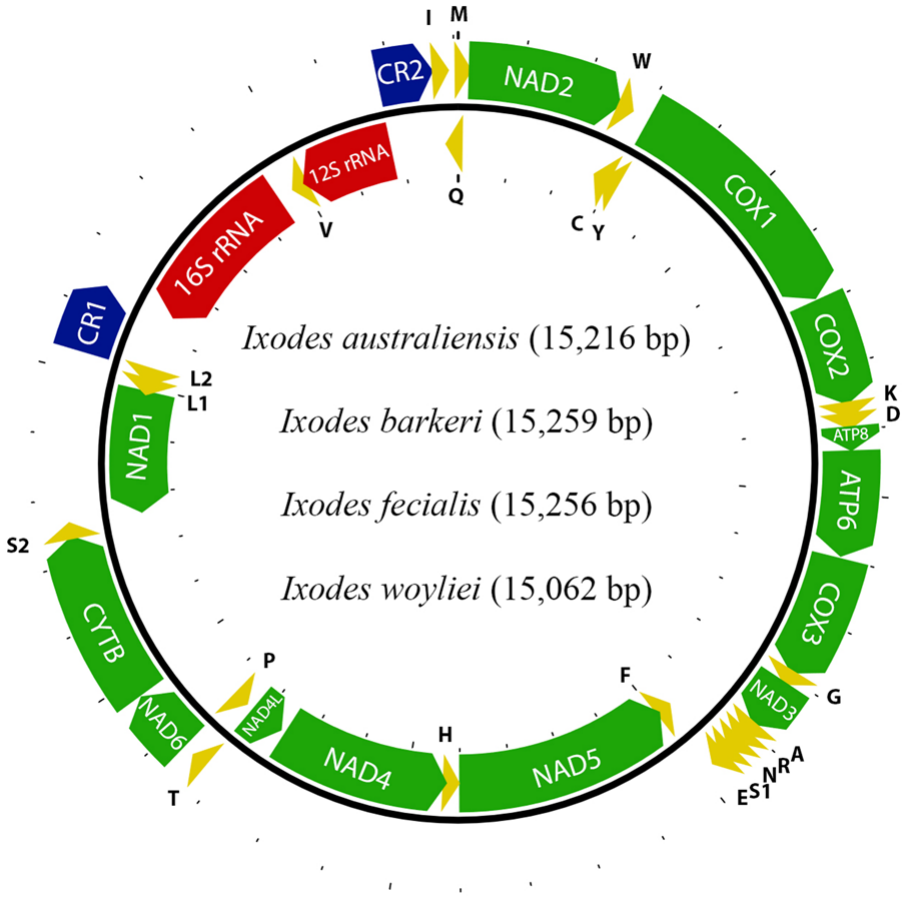
Mitochondrial genomes of *Ixodes* (*Endopalpiger*) *australiensis*, *I.* (*Endo.*) *barkeri*, *I.* (*Endo.*) *woyliei* and *I.* (*Exopalpiger*) *fecialis*. Protein-coding genes are shown in green, tRNAs are in yellow, rRNAs are in red, and the two control regions are in blue. Protein-coding genes are labelled by their four-character abbreviations, tRNAs are labelled by their one-letter amino acid abbreviations, and the two control regions are labelled as CR1 and CR2. Mitochondrial genome size variation is indicated in parentheses. The arrangement of genes in these four species is identical except that the main cluster of tRNA genes has the arrangement ARN**SE**F in the three species of *Endopalpiger* [*I.* (*Endo.*) *australiensis*, *I.* (*End.*) *barkeri* and *I.* (*End.*) *woyliei*], whereas in the one species of *Exopalpiger* [*I.* (*Exo.*) *fecialis*] the arrangement is ARN**ES**F. The arrangement in *I.* (*Exo.*) *fecialis* is the first known arrangement in an Ixodidae tick that is different from ARNSEF. Thus, ARN**ES**F might be a synapomorphy for the subgenus *Exopalpiger*

Five entire mt genomes are presented here for the first time: *I. australiensis* (OL597990, B4930, from 1 female tick), *I. barkeri* (OM302450, B5322, 1 male; OL597991, B5321, pool of 3 nymphs), *I. fecialis* (OL597993, B4928a 1 female) and *I. woyliei* (OL597992, B4928b 1 female) (Fig. 9). These mt genomes have the gene arrangement that is typical of the Australasian *Ixodes* clade ([29], Fig. 1) except that in *I. fecialis* the main cluster of tRNA genes has the arrangement ARNESF rather than ARNSEF: ARNSEF has been found in all other Ixodidae, Argasidae, Nuttalliellidae and Holothyrida studied so far [3, 4, 5, 6, 7 and Fig. 1 of 29]. Moreover, in *I. fecialis* we found a 60-bp insertion between tRNA-Asn (N) and tRNA-Glu (E) and a 57-bp insertion between tRNA-Glu (E) and tRNA-Ser (S); neither of these insertions is similar to any other motifs in the mitochondrial genome of *I. fecialis*.

**Fig. 10:**
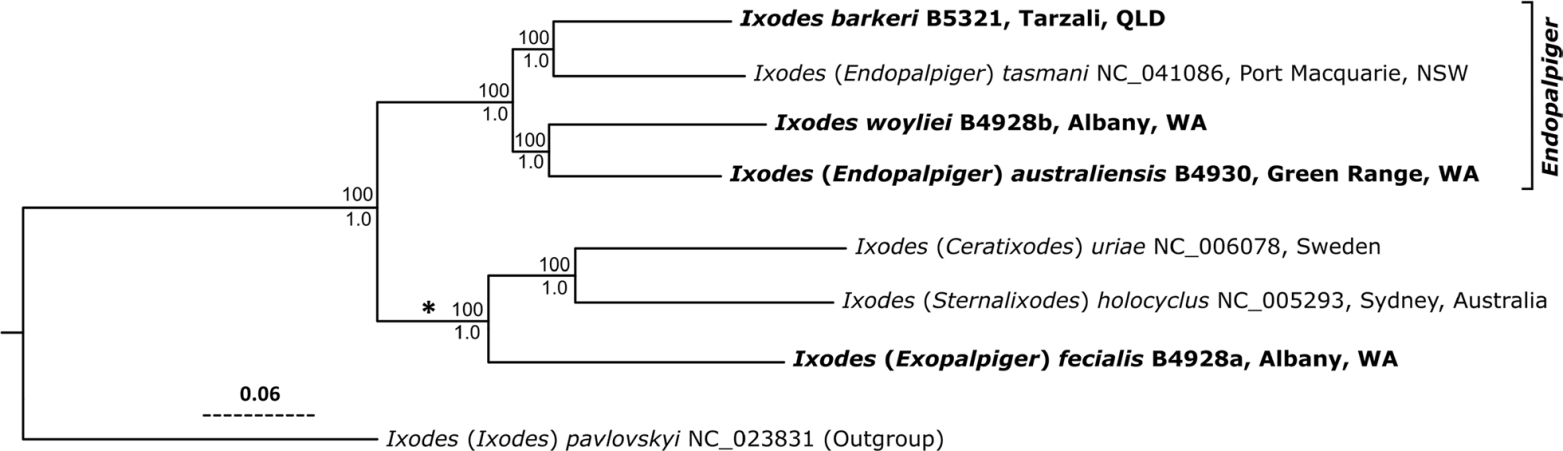
Maximum likelihood (ML) phylogenetic tree from entire mt genomes (14,935 bps). The sequence alignment was put though Gblocks to remove regions with alignment gaps. Tip labels indicate NCBI accession numbers and (Barker & Barker Collection reference nos.). Numbers above branches show maximum likelihood bootstrap support, whereas numbers below branches show the Bayesian posterior probability support. *Ixodes pavlovskyi* Pomerantzev, 1946, one of the species “Other *Ixodes*” (sensu Barker & Murrell, 2004), for which an entire mitochondrial (mt) genome was available in GenBank, was set as the outgroup. The scale bar indicates 0.06 nucleotide substitutions per nucleotide site for the 14,935 nucleotide sites in our alignment of theses entire mt genomes. So, for example, there were about 896 nucleotide substitutions along the branch that leads to *I.* (*Ceratixodes*) *uriae* plus *I.* (*Sternalixodes*) *holocyclus* plus *I.* (*Exopalpiger*) *fecialis*, which is marked with an asterisk [i.e. 0.06 nucleotide substitutions per nucleotide site × 14,935 nucleotide sites (bps) = 1896 nucleotide substitutions]. Ticks in bold were sequenced in the present study

The phylogeny from these mt genomes indicates that *I. barkeri* and *I*. *woyliei* are best placed in the subgenus *Endopalpiger* Schulze, 1935, since *I. barkeri* and *I*. *woyliei* were in a lineage with *I.* (*Endopalpiger*) *australiensis* and *I*. (*Endopalpiger*) *tasmani* to the exclusion of species from the subgenera *Ceratixodes, Exopalpiger* and *Sternalixodes*: *I.* (*Ceratixodes*) *uriae*, *I*. (*Exopalpiger*) *fecialis* and *I.* (*Sternalixodes*) *holocyclus* (Fig. 10).

## Discussion

We had hoped to extract sufficient DNA for our experiments from all of the putative life-stages of *I. barkeri* [1] i.e. females, males, nymphs and larvae. Adequate DNA for our experiments, however, was obtained from only a male and a pool of three nymphs but not from females nor larvae since the females and larvae had not been preserved well enough. The mt genomes sequences of the male (GenBank OM302450) and the pool of three nymphs (OL597991) were > 99.96 % identical; thus, the nymphs were certainly *I. barkeri* [1]. Although, we do not have mt genome sequences from the females or the larvae the evidence that the females and larva are also attributable to *I. barkeri* [1] is strong on account of the morphological similarity of the females and larvae to the males and nymphs (above and Figs. 2, 3, 4, 5, 6, 7, 8, 9). The subgeneric classification of *Ixodes* is complex and sometimes ignored, probably because some subgenera are defined ambiguously, making species difficult to place in a subgenus. However, the names of the subgenera are valid, represent hypotheses of relationships and deserve closer attention. Previously, neither [8] nor [1] attempted to place *I. woylie* and *I. barkeri*, respectively, in a subgenus. We, however, conclude that *I. woylie* and *I. barkeri* are best placed in the subgenus *Endopalpiger* Schulze, 1935 (Fig. 10). Alas, mt genomes from the other species of *Endopalpiger* were not available to us: (i) *I. victoriensis* Nuttall, 1916, and *I. hydromyidis* Swan, 1931, from Australia; (ii) *I. acer* Apanaskevich, 2020; *I. giluwensis* Apanaskevich 2020*; I. luxuriosus* Schulze, 1935; *I. mirzai* Apanaskevich, 2020*; I. planiscutatus* Apanaskevich, 2020*; I. steini* Schulze, 1935; *I. stellae* Apanaskevich, 2020; and *I. zaglossi* Kohls, 1960, from Papua New Guinea.

Paul Schulze was a prolific German taxonomist whose life works were reviewed recently [26]. He described 17 entities that are presently considered as subgenera [30], including *Endopalpiger* in 1935, with *Ixodes luxuriosus* Schulze, 1935, as the type species (redescribed by [20]). The subgenus *Endopalpiger* was based mainly on their prominent and distinctive palps. Later, Schulze [31] gave generic status to *Endopalpiger*, thus emphasizing the very unusual form of the palps. [32] and [19] considered the subgenus *Endopalpiger* to be valid, but [33] and [34] presented the subgenus *Endopalpiger* as a synonym of *Exopalpiger* Schulze, 1935, but without evidence or argument. Here, our phylogenetic trees show that *Endopalpiger* and *Exopalpiger* are not closely related. Rather, *Exopalpiger* is much closer to *Sternalixodes* and *Ceratixodes* than it is to *Endopalpiger* (Fig. 10).

The four species of *Endopalpiger* in our tree formed a monophyletic group (*barkeri, tasmani, woylie, australiensis*); indeed, a monophyletic group with 100% bootstrap support and a posterior probability of 1.0, the highest possible posterior probability (Fig. 10). This is the first phylogenetic tree from entire mt genomes (about 15,000 bps) or any similarly large number of nucleotides. [The only other tree that had more than one species of *Endopalpiger* was by [8] (Fig. 10; ca. 800 bps of *cox1*)]. Therefore, we found strong support for *Endopalpiger*, albeit with a limited set of taxa. The unique nature of palpal segment (article) I is a morphological synapomorphy of *Endopalpiger*. As described by [19] (p. 13), the female palpal segment I (“I” in Fig. 5C) is greatly enlarged and projects inwardly and forwardly so that it *ensheathes* each side of the base of the mouthparts, and ventrally palpal segment I is strongly salient (“ss” in Fig. 5C). The only similar palp morphology in adults is that of *Exopalpiger*, which, in the words of [19] (p. 13), sounds more like that of *Endopalpiger* than it actually is. According to [19] (p. 13), the female palpal segment 1 of *Exopalpiger* is also greatly enlarged, being attached at right angles to the transverse axis of the basis, but does not project inwardly or forwardly and it does *not ensheathe* any part of the base of the mouthparts; ventrally palpal segment I is salient but not as salient as in *Endopalpiger.*

## Data Availability

The data supporting the conclusions of this article are in the article. The mitochondrial genomes published for the first time in this paper have been submitted to GenBank database, accession numbers  OL597990-OL597993, and OM302450.

## Abbreviations

ANIC: Australian National Insect Collection
mt: Mitochondrial
